# Comparison of marginal fit of cemented zirconia copings manufactured after digital impression with lava™ C.O.S and conventional impression technique

**DOI:** 10.1186/s12903-016-0323-8

**Published:** 2016-12-08

**Authors:** Rinet Dauti, Barbara Cvikl, Alexander Franz, Uwe Yacine Schwarze, Bledar Lilaj, Tina Rybaczek, Andreas Moritz

**Affiliations:** 1 Department of Conservative Dentistry and Periodontology, Medical University of Vienna, Sensengasse 2A, 1090 Vienna, Austria; 2 Department of Preventive, Restorative and Pediatric Dentistry, School of Dental Medicine, University of Bern, Bern, Switzerland; 3 Karl Donath Laboratory for Hard Tissue and Biomaterial Research, University Clinic of Dentistry, Vienna, Austria; 4 Department of Oral Biology, Medical University of Vienna, Vienna, Austria; 5 Department of Oral Surgery, Medical University of Vienna, Vienna, Austria

**Keywords:** Marginal gap, Digital impression, Lava C.O.S, Zirconia copings

## Abstract

**Background:**

Evaluation of the marginal fit of cemented zirconia copings manufactured after digital impression with Lava™ Chairside Oral Scanner in comparison to that of zirconia copings manufactured after conventional impressions with polyvinyl siloxane.

**Methods:**

A prepared typodont tooth #36, was replicated 40 times with a vinyl silicone and precise model resin. The dies were randomly divided into two groups according to the impression taking technique. Digital impressions with Lava™ C.O.S. and conventional impressions were taken according to the group. Subsequently zirconia copings were manufactured and cemented on their respective dies with zinc oxide phosphate cement. After embedding in resin, mesio-distal section of each coping was performed with a diamond saw in order to obtain two slices. One half of the specimen was used for evaluation with an optical microscope (OM) and the other half for evaluation with a scanning electron microscope (SEM). Marginal gap (MG) and absolute marginal discrepancy (AMD) were measured mesial and distal on each slice.

**Results:**

No significant difference of the marginal parameters between the digital and the conventional group was found. The mean values for MG in the digital group were 96.28 μm (+/−43.21 μm) measured with the OM and 99.26 μm (+/−48.73 μm) measured with the SEM, respectively. AMD mean values were 191.54 μm (+/−85.42 μm) measured with the optical microscope and 211.6 μm (+/−96.55 μm) with the SEM. For the conventional group the mean MG values were 94.84 μm (+/−50.77 μm) measured with the OM and 83.37 μm (+/−44.38 μm) measured with the SEM, respectively. AMD mean values were 158.60 μm (+/−69.14 μm) for the OM and 152.72 μm (+/−72.36) for the SEM.

**Conclusions:**

Copings manufactured after digital impression with Lava™ C.O.S. show comparable marginal parameters with the copings manufactured after conventional impression with polyvinyl syloxane. The mean MG values of both groups fit in the clinically acceptable range.

**Electronic supplementary material:**

The online version of this article (doi:10.1186/s12903-016-0323-8) contains supplementary material, which is available to authorized users.

## Background

The impression taking procedure is an essential step in the manufacturing process of dental restorations [1]. It provides a link between the dentist’s and the dental technician’s work for an exact reproduction in the clinical situation. Precision in the impression procedure is a prerequisite for accurate casts and subsequently for the precise fitting of restorations [2]. Beside the conventional technique in which diverse impression materials are used, currently the digital impression technique is also available [3].

Conventional impression techniques require no special expensive machinery and accurate results can be achieved if working steps are conducted properly [4]. Impression materials frequently used for this technique are polyvinyl siloxane, polyether or polysulfide based materials. For attaining a perfect cast these materials must demonstrate properties like accuracy, elastic recovery and dimensional stability as well as rheological and thixotropic characteristics [5]. Various factors like uncontrolled saliva flow during the procedure, undercuts, storing for extended periods of time, moisture, material deformations and incompatibilities with other materials can influence the accuracy of the impression and subsequently lead to inaccuracies and misfit of restorations [6].

The goal of the initial developments in computer aided design and in the computer aided manufacturing (CAD/CAM) technologies was to achieve digital intraoral impression techniques that simplify the production process, reduce costs and improve patient comfort [7]. The reduction of both intermediate production steps and possible sources of error is of particular clinical importance [8]. Furthermore digital intraoral impression allows for a fully digitalized production workflow [9]. Working steps such as tray selection, tray try out, impression disinfection, transportation, plaster pouring, trimming or articulation can be omitted [10]. Moreover it facilitates not only real-time imaging and chair-side analysis of the preparation but also selective scanning of particular areas, digital archiving and faster communication with the dental lab [11, 12]. In the digital production process the preparation is captured by the acquisition unit and converted into geometrical digital data [13]. The subsequent data processing results in a virtual model of the prepared tooth. Based on this virtual model the restoration is first designed and then fabricated in the CAM unit [14]. Applying this digital workflow in clinical practice introduces new working steps for both dentist and dental technician that possibly influence the accuracy of the scan and ultimately of the fit of the restorations [15].

The marginal fit in restorations is of utmost importance for their quality and longevity, and was one of the main initial concerns of the CAD/CAM systems [16]. The marginal fit is theoretically represented by a linear contact line or a gap-free transition between the preparation and the restoration margin. For clinical use, Christensen et al. concluded that visible margins wider than 39 μm are clinically unacceptable [17]. However, due to various factors, it seems almost impossible to achieve these ideal values in the clinical setting [18, 19]. According to literature, a marginal gap between 50 and 100 μm is considered to be technically feasible [20]. Larger marginal gaps would provide a niche for oral pathogens and saliva, leading to problems like periodontal inflammations, secondary caries as well as cement dissolution [21, 22].

Marginal fit that was considered to be clinically sufficient was achieved when using digital, intraoral impression procedures with the Lava™ Chairside Oral Scanner (Lava™ C.O.S., 3 M ESPE, Seefeld, Germany) [23]. However to our knowledge, there are no studies on the marginal fit of cemented zirconia copings using digital, intraoral impression procedures with the Lava™ C.O.S. in comparison to conventional impression procedures. Therefore we investigated the marginal fit of cemented zirconia copings manufactured after digital impression with Lava™ C.O.S. in comparison to conventional impressions with polyvinyl siloxane. The analyses are based on optical microscope pictures and scanning electron microscope pictures, respectively. Furthermore, the results gained by the optical microscope and the scanning electron microscope were compared.

## Methods

**Fig. 1:**
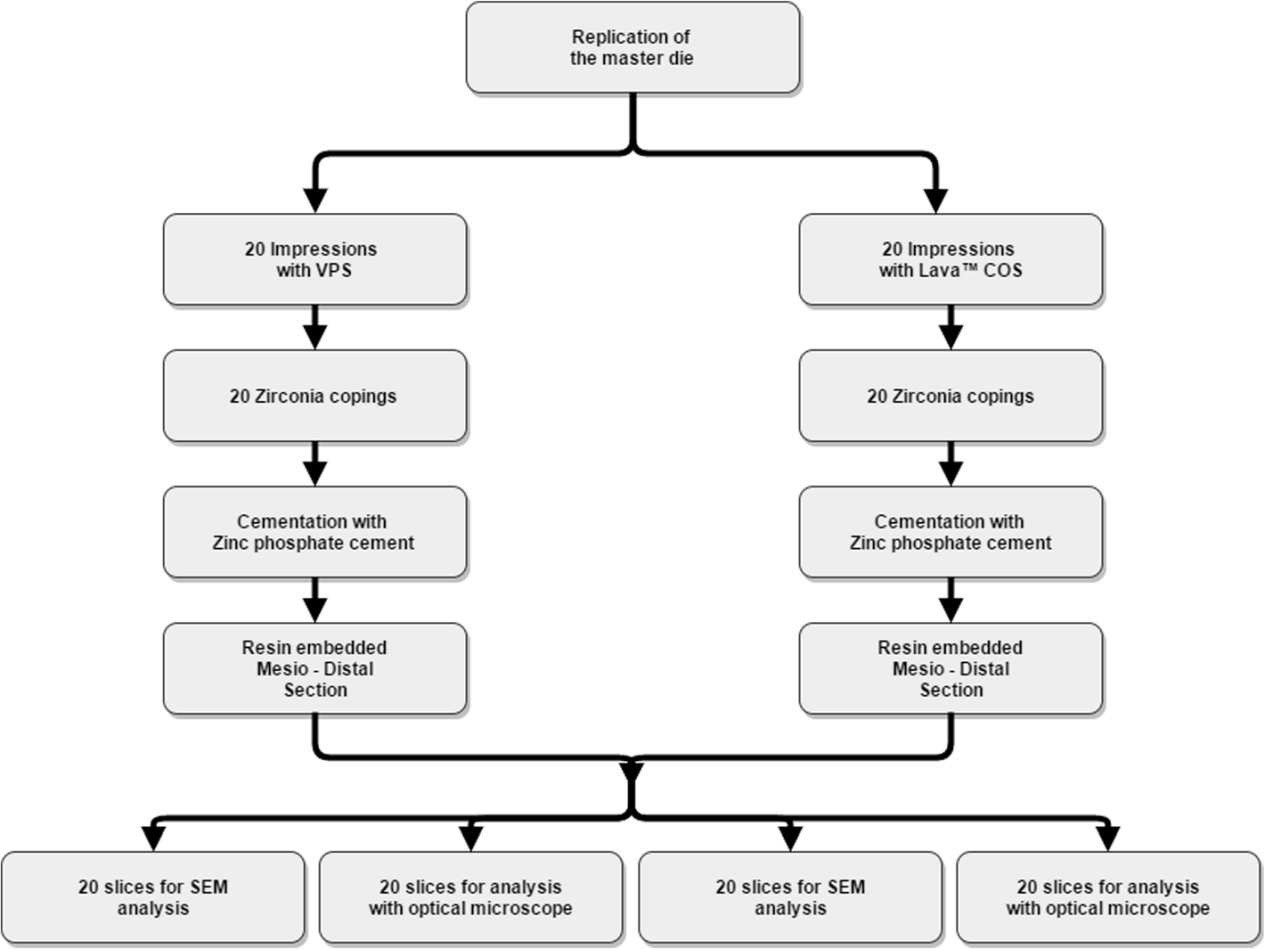
Experimental design

The aim of this in vitro study was to investigate the marginal fit of cemented zirconia copings manufactured after digital impression with Lava™ C.O.S. in comparison to conventional impressions with polyvinyl siloxane. A flowchart of the experimental procedures is given in Fig. 1. One typodont plastic tooth (left first molar, KaVo Dental, Biberach/Riß, Germany) was prepared with a circumferential reduction of 0.8–1.2 mm, occlusal reduction of 1.5 mm, chamfer finish line of 0.8 mm and convergence angle of the axial walls of 6°. The master die was replicated 40 times using a high quality vinyl silicone (DUOSIL D, SHERA Werkstoff-Technologie, Lemförde, Germany) and highly precise model resin (Mirapont^®^, Hager & Werken, Duisburg, Germany). Afterwards, the replicated dies were randomly divided into two groups according to the impression-taking technique (digital or conventional). The dies of each group were fixed in plaster blocks (five dies in each block) and marked in order to facilitate the identification in further steps.

Conventional impressions were taken with a polyvinyl siloxane impression material (Imprint II Garant®, 3 M ESPE, Seefeld, Germany) in a one-step technique using individual trays fabricated with cold-curing material (SR Ivolen, Ivoclar Vivadent, Liechtenstein). The impressions were inspected by the same operator under a microscope at 10× magnification (Stemi DV4 Spot, Carl Zeiss Microscopy, Jena, Germany) and then poured with a type IV plaster (SHERAHARD-ROCK, SHERA Werkstoff-Technologie, Lemförde, Germany). From the plaster models a saw-cut model was fabricated and each of the plaster model dies was individually digitalized with a laboratory dental scanner (3Shape Scanner D700, Wieland Dental + Technik, Pforzheim, Germany).

Digital impressions were taken with the Lava™ C.O.S. (Software version 3.0.2), an intra-oral digitizing system that creates the impressions by means of continuous 3D video images. These video images are possible due to three sensors that simultaneously capture the dies from three different perspectives. The dies were slightly dusted with titanium oxide powder for optical scanning (Lava™ Powder for Chairside Oral Scanner, 3 M ESPE, Seefeld, Germany) with the corresponding sprayer (Lava™ Sprayer, 3 M ESPE, Seefeld, Germany) directly before the scanning process. The data sets were sent to the company 3 M ESPE via Internet and were made available for download in the Lava C.O.S. Lab Software (3 M ESPE, Seefeld, Germany) 24 h later. The dies were then virtually cut and marked.

Scanned data from the digital and conventional group were transmitted to the 3Shape DentalDesigner™ software (Wieland Dental + Technik, Pforzheim, Germany) in order to design the copings. The marginal fitting parameters were set to 0.01 mm thickness to a level of 1 mm above the margin and the cement space was set to 0.04 mm. The data of the virtually constructed copings were then transferred into the Zenotec CAM basic software V 2.2.17 (Wieland Dental + Technik, Pforzheim, Germany) for computation of the milling paths and the milling strategies. The milling process took place in a ZENOTEC mini milling machine (Wieland Dental + Technik, Pforzheim, Germany) from a Zenostar Zr Translucent blank (Wieland Dental + Technik, Pforzheim, Germany). The enlarged copings were sintered in a Cercon® heat plus furnace (DeguDent GmbH, Germany) for 8 h at 1350 °C. The copings of both groups were placed on their respective dies and checked for irregularities under a microscope at 10x magnification (Stemi DV4 Spot, Carl Zeiss Microscopy, Jena, Germany).

For cementation, the copings were seated on their respective dies using zinc oxide phosphate cement (HOFFMANN’S CEMENT quick setting, Hoffmann Dental Manufaktur, Berlin, Germany) after try on. The copings were held under constant finger pressure for 10–15 min until the cement was set. The cement was mixed to the manufacturer’s specified powder/liquid ratio with a spatula on the rough side of a pre-cooled mixing glass slab to a fixation consistency. After removing the cement residues from the margins the dies were detached from the plaster blocks.

After 24 h all specimens were dehydrated and degreased in absolute ethanol for 2 days and subsequently infiltrated with a light-curing resin (Technovit 7200®, Kulzer & Co., Wehrheim, Germany) in ascending grades of ethanol/resin mixtures. Finally the specimens were embedded in pure resin. Each embedded specimen was cut in mesio distal direction with a diamond-coated band saw (EXAKT 300, Exakt, Norderstedt, Germany) for obtaining two parts of the same size.

**Fig. 2:**
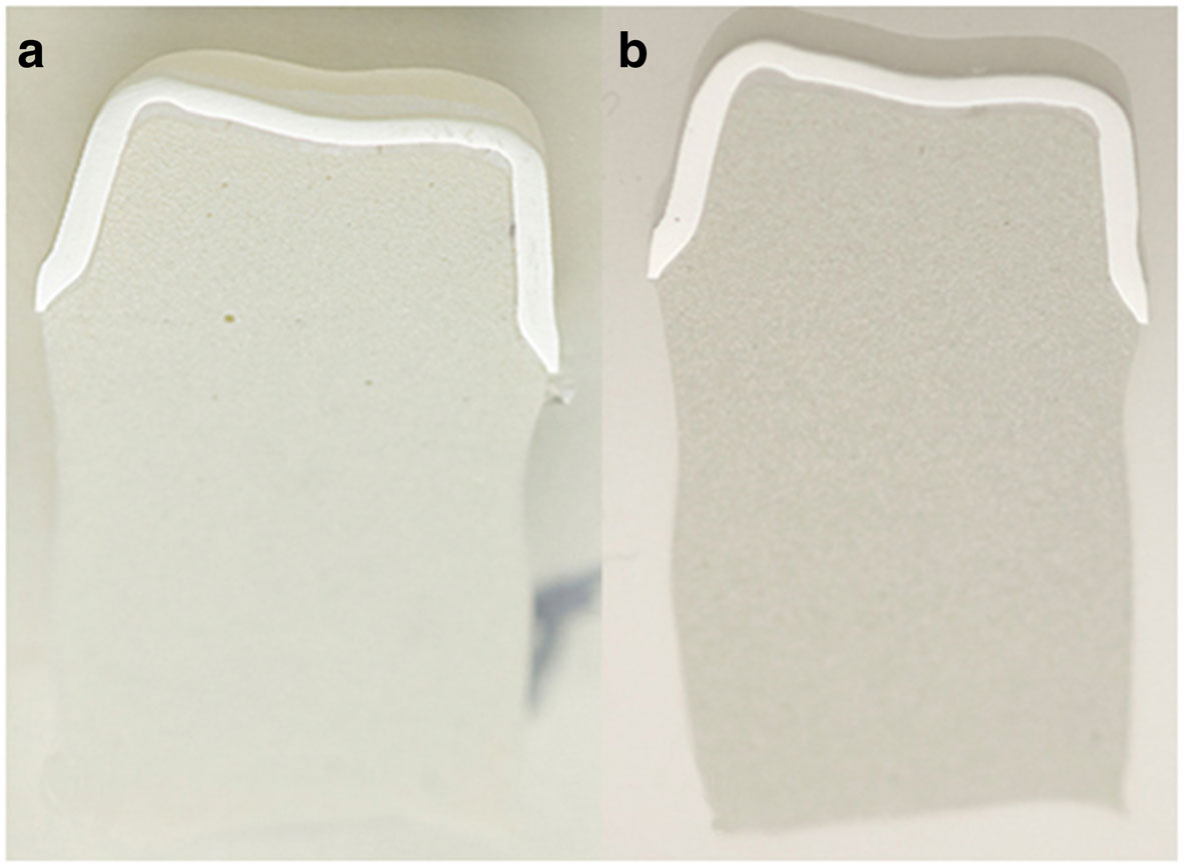
**a** Slice for SEM measurements. **b** Slice for optical microscope measurements

Thin ground sections of approximately 30 μm thickness were prepared from one half of the specimen using the cutting-grinding technique of Donath [24] for the measurements using the optical microscope (Olympus BX51, Olympus, Japan) Fig. 2b). Images with a resolution of 0.16 μm per pixel were captured on 40x magnification using the Olympus dotSlide 2.4 - digital microscopy system (Olympus, Tokyo, Japan).

The remaining half of the dissected ceramic specimens was prepared for measurements using the TM-1000 tabletop scanning electron microscope; Hitachi, Krefeld, Germany Fig. 2a). Images in 200×, 300×, and 400× magnification were taken and analyzed using the Hitachi’s SEM software Ver. 03–02.

**Fig. 3:**
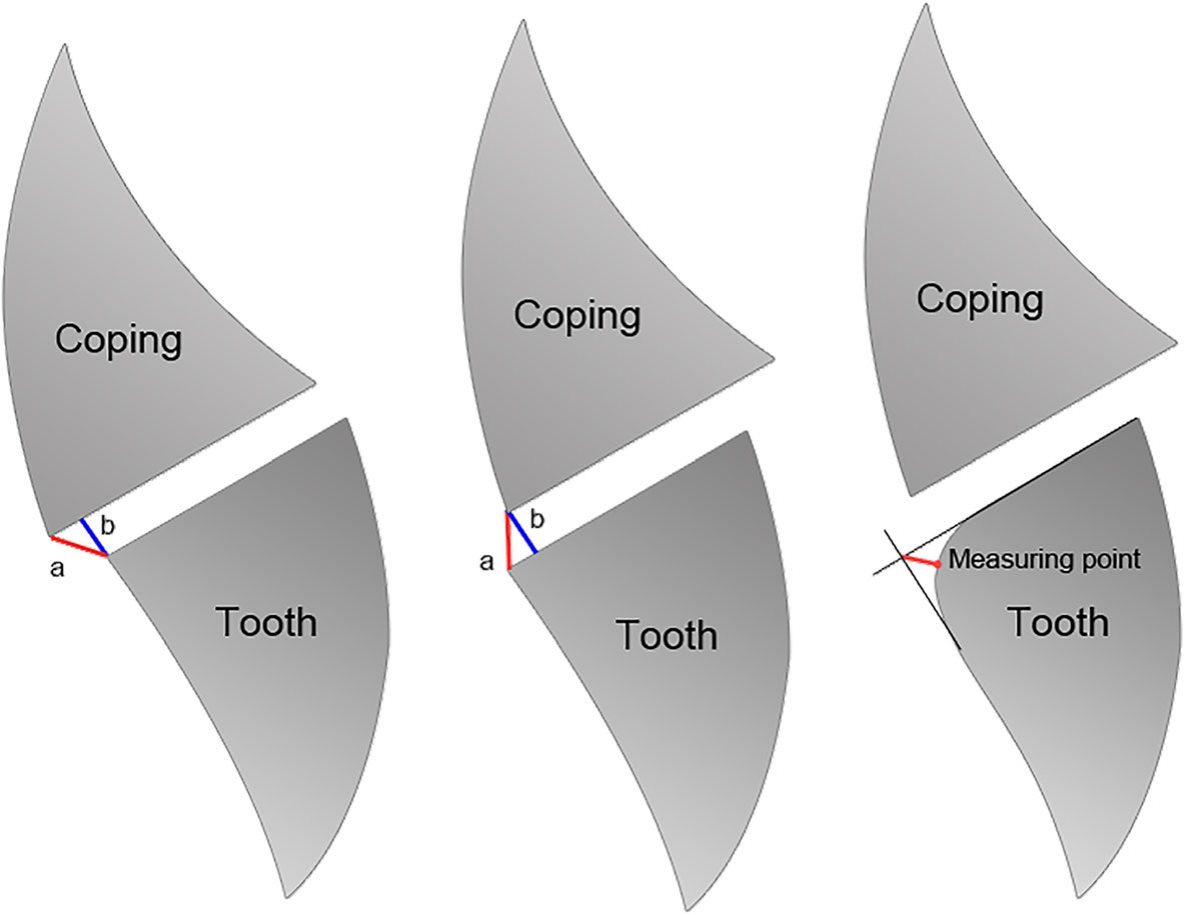
**A** AMD (a) and MG (b) on overextended copings **B** AMD (a) and MG (b) on underextended copings **C** Measuring point on rounded margins

Marginal gap (MG) (Fig. 3A (b) and B (b)) and the absolute marginal discrepancy (AMD) (Fig. 3A. (a) and B (a)), were measured in accordance with Holmes et al. [25]. Marginal gap is defined as the perpendicular distance between the internal surface of the restoration and the preparation line for overextended copings or the perpendicular distance from the restoration margin to the tooth surface for underextended copings. The absolute marginal discrepancy represents the distance between the restoration margin and the preparation line. By rounded preparation margins the measuring point was determined by extending the main contours of the die and drawing an angle bisector as shown in Fig. 3c.

**Fig. 4:**
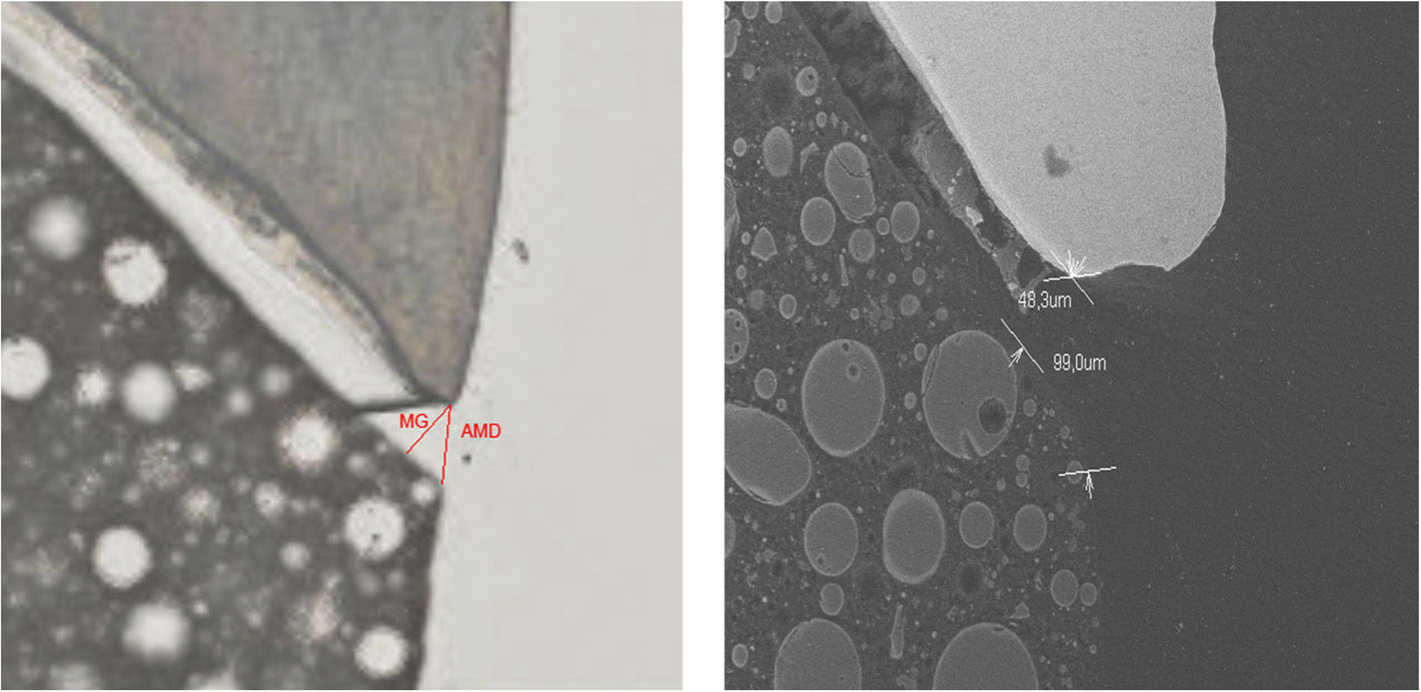
**a** Measurement with optical microscope. **b** Measurement with SEM

For both groups on each specimen MG and AMD were measured with an optical microscope and SEM on the mesial and distal aspect (Fig. 4). The values for either MG or AMD for the mesial and distal aspect of each slice were summed up and mean values for MG and AMD were calculated. Consequently, the groups were as follows: i) AMD OM, ii) AMD SEM, iii) MG OM and iv) MG SEM.

Five additional dies and their respective copings were manufactured in order to compare the results of MG and AMD when another technique, namely the replica technique was used. For this a light body silicone impression material (Imprint II Garant®, Light Body) was used to fill the copings before they were seated on the respective dies by applying constant finger pressure simulating the cementation process. After five minutes the copings were removed together with the adhered impression material, which indicates the gap between die and coping. To be able to perform the measurements of MG and AMD a regular body material with a different colour (Imprint II Garant®, Regular Body) was placed in the copings. After setting the silicone replicas were removed, sectioned in a mesio distal direction and the MG and the AMD were measured using the optical microscope. Since the replica technique is a non-destructive method for both, the dies and the copings, MG and AMD could be also measured after cementation using the zinc oxide phosphate cement as described above.

For each of the measurements (AMD OM, AMD SEM, MG OM and MG SEM) Student’s *t*-tests between the digital and conventional technique were performed. Statistical outcome was adjusted for multiple testing using Holm’s method [26]. Intraclass correlation coefficient (ICC) was used to assess agreement of SEM and optical measurements. R 2.15.1 (R Core Team 2012) and ggplot2 0.9.2.1 were used for all computations.

## Results

Altogether 306 measurements were collected. 14 measuring locations were excluded (6 from the conventional and 8 from the digital group) because of damages on the slice surface.

The mean values for MG in the digital group were 96.28 μm measured with the optical microscope and 99.27 μm measured with the SEM, respectively. AMD mean values were 191.54 μm measured with the optical microscope and 211.6 μm with the SEM.

**Fig. 5:**
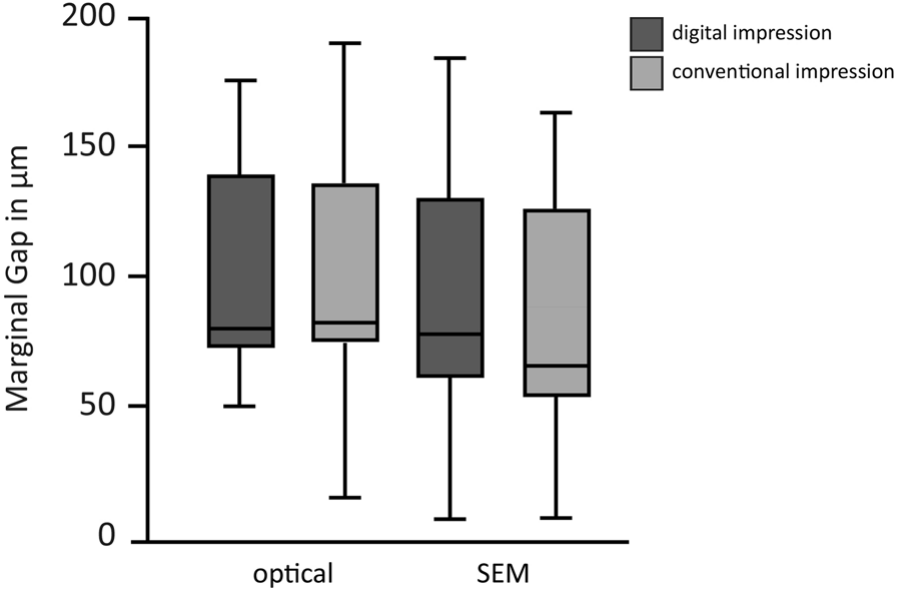
Median and standard deviations of MG. Values in μm

**Fig. 6:**
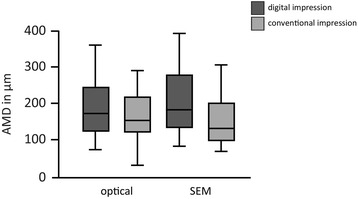
Median and standard deviations of AMD. Values in μm

For the conventional group the mean MG values were 94.85 μm measured with the optical microscope and 83.38 μm measured with the SEM, respectively. AMD mean values were 158.61 μm for the optical microscope and 152.72 μm for the SEM (Tables 1 and 2). Median and standard deviations values are presented in Figs. 5 and 6.

**Table 1:** Mean values and standard deviations of MG in both groups in μm

Group	Microscope	Mean	SD
Lava™ C.O.S.	MG Optical	96.283	43.213
Convent.	MG Optical	94.845	50.773
Lava™ C.O.S.	MG SEM	99.265	48.737
Convent.	MG SEM	83.376	44.381

**Table 2:** Mean values and standard deviations of AMD in both groups in μm

Group	Microscope	Mean	SD
Lava™ C.O.S.	AMD Optical	191.543	85.422
Convent.	AMD Optical	158.609	69.147
Lava™ C.O.S.	AMD SEM	211.600	96.557
Convent.	AMD SEM	152.721	72.364

*P*-values are presented in Table 3. No significant difference was found between the digital and conventional group, neither in the optical nor in the SEM. The intra class correlation coefficient value was 0.921.

**Table 3:** Holm adjusted *p*-values of Student’s t-tests for both groups

Groups		*p*-values
MG SEM	Dig. vs conv.	0.614
MG Optical	Dig. vs conv.	0.925
AMD SEM	Dig. vs conv.	0.166
AMD Optical	Dig. vs conv.	0.611

Additional experiments for comparing the replica technique with the cementation technique showed median values of 91.2 μm and 185.3 μm, respectively for MG and AMD when the replica technique was used. The same specimens showed median values of 70.2 μm and 122.8 μm, respectively for MG and AMD when the cementation technique was used.

## Discussion

Digital intraoral optical impressions, as well as other digital applications are advancing in the field of restorative dentistry and are becoming a serious alternative to the conventional method [11]. The aim of this study was to evaluate and compare the fit of cemented zirconia copings manufactured after a digital impression with Lava™ C.O.S. with copings manufactured after conventional impression. In this study the measurements of the marginal gap (MG) and of the absolute marginal discrepancy (AMD) with an optical microscope and a SEM showed no significant difference between the digital and the conventional impression technique group. Furthermore mean MG values of the copings for both groups were smaller than 100 μm, showing clinically acceptable margins [20]. Calculation of the intra class correlation coefficient showed a relatively high value of 0.921, which suggests similarity of measurements with the optical and the SE-microscope.

It is difficult to compare the results of this study with recent literature due to differences in measuring techniques and measuring parameters used for the evaluation of fit of fixed dental restorations. An in vivo study evaluating the Lava™ C.O.S. showed significantly better fit of crowns in favor of the digital impression. Median marginal gap was 49 μm compared to 74 μm for the conventional group [23]. Compared to the abovementioned study, in the present in vitro trial the median marginal gap for the digital group was 79.57 μm measured with the optical microscope and 88.02 μm with the SEM. First thoughts were that the main reason for the elevated values in the present study was the cementation of the copings with zinc phosphate cement, which might have elevated the marginal parameters [20] especially since the measurements in the abovementioned studies were performed using the silicone replica method. The results of fit using this method depend on the viscosity of the selected silicone material and cannot totally reproduce the thickness of ZnO phosphate cement [27]. In order to verify this idea additional experiments were performed comparing the marginal parameters when the replica technique was used with the cemented copings. Contrary to expectations, MG and AMD showed similar results when using the replica technique and the cementation technique. Nevertheless this is in accordance with studies by Rahme et al., showing that the marginal fit was similar after using the replica technique and after cementation with glass-ionomer cement [28]. Another study investigating inter alia the marginal fit after using the replica technique compared to cemented samples also showed no significant differences, reporting values of about 100 μm independent of the technique, which is also in accordance with the values of the present study [29].

In another in vitro study a travelling microscope and digital micrometer heads were used to evaluate the influence of conventional and digital impressioning techniques on the accessible marginal inaccuracies (AMI) of crowns [9]. Crowns manufactured after digital impression with Lava™ C.O.S. showed an AMI value of 48 (±25) μm, which is in accordance with the studies named above. In a further study 3D and 2D assessment of the marginal gap was conducted on crowns produced after conventional and digital impression with Lava™ C.O.S [30]. A new method with a precise scanner and best fitting algorithm software was used for the measurements. The Lava™ C.O.S. group showed mean 3D values for MG of 84 μm and mean 2D values of 74 μm, similar to the MG results of the presented study, which are reported to fit the clinical acceptable range [20]. The higher values of the AMD could be explained by the fact that the AMD reflects the total misfit, which also includes horizontal discrepancies, while the MG only measures the vertical discrepancy [31].

The impressions and the manufacture of the copings in the present study were performed under laboratory conditions, so the influence of difficulties such as subgingival preparation margins, blood and saliva contamination, or the reaction of the patient to the digital or conventional impression cannot be taken into consideration. Furthermore the marginal gap (MG) and the absolute marginal discrepancy (AMD) measured on the copings in this study are not a direct evaluation of the accuracy of the impression method but rather an examination of the whole working process from preparation up to cementation. Possible errors on each part of the manufacturing process, such as setting of the milling parameters, designing and sintering shrinkage have direct influence on the marginal fit. In order to simulate the clinical situation the cement mixing procedure and the cementation of the copings were performed manually accepting a non perfectly standardized procedure. Ditto for the replica technique constant finger pressure was used to seat the copings on the respective dies.

Another limitation of our study was the section of the copings in the mesio-distal direction, which made available only two points of the circumference of the die for the measurement process. Thus, besides the samples were destroyed and not available for repeated measurements it was not possible to evaluate the level of fit at the whole circumference whereby larger values of the MG and AMD could be missed. Therefore further studies visualizing and measuring the whole marginal and cement gap using non-invasive methods such as micro computed tomography or virtual fit assessment should be performed.

## Conclusion

Within the limitations of this in vitro study we can conclude that:

1. Copings manufactured after digital impression with Lava™ C.O.S. show comparable marginal parameters with the copings manufactured after conventional impression with polyvinyl syloxane.
2. The mean marginal gap values of the digital and the conventional group fit in the clinically acceptable range.

## Additional file

Additional file 1: Dauti et al._raw data. Description of data: Results showing MG and AMD in mesial and distal position for the digital and conventional group measured with the SEM and the optical microscope. Furthermore the results after additional experiments using the silicone replica technique are shown. (PDF 31 kb)

## Abbreviations

AMD: Absolute marginal discrepancy
CAD: Computer aided design
CAM: Computer aided manufacturing
Lava™ C.O.S.: Lava™ chairside oral scanner
MG: Marginal gap
OM: Optical microscope
SEM: Scanning electron microscope
