# Supplementary material for: Comparison of marginal fit of cemented zirconia copings manufactured after digital impression with lava™ C.O.S and conventional impression technique
Source: BMC Oral Health. 2016 Dec 8;16:129. doi: 10.1186/s12903-016-0323-8 (PMC5146899; doi:10.1186/s12903-016-0323-8)
Supplement: Additional file 1: — Dauti et al._raw data. Description of data: Results showing MG and AMD in mesial and distal position for the digital and conventional group measured with the SEM and the optical microscope. Furthermore the results after additional experiments using the silicone replica technique are shown. (PDF 31 kb) [file 12903_2016_323_MOESM1_ESM.pdf]

Slice nr.	AMD	MG	Position	Group	Microscope
4434	43,2	11,2	mesial	conventional	SEM
4434	89,7	42,1	distal	conventional	SEM
4435	47,3	33,2	mesial	conventional	SEM
4435	101	74,1	distal	conventional	SEM
4436	31	17	mesial	conventional	SEM
4436	180	85,8	distal	conventional	SEM
4437	64,4	52,5	mesial	conventional	SEM
4437	88,7	34	distal	conventional	SEM
4438	114,6	11,7	mesial	conventional	SEM
4438	203	57,5	distal	conventional	SEM
4549	262	217	mesial	conventional	SEM
4549	221	54,9	distal	conventional	SEM
4550	284	245	mesial	conventional	SEM
4550	196	65,6	distal	conventional	SEM
4551	not available	not available	mesial	conventional	SEM
4551	123	110	distal	conventional	SEM
4552	not available	not available	mesial	conventional	SEM
4552	132	81,7	distal	conventional	SEM
4553	not available	not available	mesial	conventional	SEM
4553	297	208	distal	conventional	SEM
4640	202	154	mesial	conventional	SEM
4640	431	168	distal	conventional	SEM
4641	312	84,4	mesial	conventional	SEM
4641	36,7	36,7	distal	conventional	SEM
4642	53,7	52,6	mesial	conventional	SEM
4642	195	111	distal	conventional	SEM
4643	58,8	48	mesial	conventional	SEM
4643	191	91,4	distal	conventional	SEM
4644	182	121	mesial	conventional	SEM
4644	219	126	distal	conventional	SEM
4645	98,7	66,6	mesial	conventional	SEM
4645	92,3	63,8	distal	conventional	SEM
4646	78,5	55,1	mesial	conventional	SEM
4646	119	86,4	distal	conventional	SEM
4647	80	47,9	mesial	conventional	SEM
4647	110	43,6	distal	conventional	SEM
4648	132	106	mesial	conventional	SEM
4648	224	74,9	distal	conventional	SEM
4649	376	279	mesial	conventional	SEM
4649	74,9	16,8	distal	conventional	SEM
4434	20,8	18,7	mesial	conventional	optical
4434	37,94	17,5	distal	conventional	optical
4435	38,31	35,56	mesial	conventional	optical
4435	160,41	88,45	distal	conventional	optical
4436	54,09	34,75	mesial	conventional	optical
4436	121,13	51,25	distal	conventional	optical
4437	44,86	42,2	mesial	conventional	optical
4437	65,34	39,32	distal	conventional	optical

4438	93,96	37,17	mesial	conventional	optical
4438	174,73	6,51	distal	conventional	optical
4549	275,93	229,45	mesial	conventional	optical
4549	138,7	68,42	distal	conventional	optical
4550	335,81	222,7	mesial	conventional	optical
4550	129,71	39,37	distal	conventional	optical
4551	345,8	259,97	mesial	conventional	optical
4551	146,03	121,64	distal	conventional	optical
4552	192,16	164,4	mesial	conventional	optical
4552	122,99	96,74	distal	conventional	optical
4553	148,25	106,98	mesial	conventional	optical
4553	309,45	226	distal	conventional	optical
4640	316,3	181,46	mesial	conventional	optical
4640	189,7	102,5	distal	conventional	optical
4641	148,73	78,3	mesial	conventional	optical
4641	94,45	76,06	distal	conventional	optical
4642	67,05	62,11	mesial	conventional	optical
4642	203,52	97,14	distal	conventional	optical
4643	80,19	75,42	mesial	conventional	optical
4643	234,4	89,73	distal	conventional	optical
4644	190,17	44,52	mesial	conventional	optical
4644	223,46	134,31	distal	conventional	optical
4645	153,4	81,93	mesial	conventional	optical
4645	167,94	77,59	distal	conventional	optical
4646	51,41	42,66	mesial	conventional	optical
4646	173,65	99,12	distal	conventional	optical
4647	160,26	84,97	mesial	conventional	optical
4647	78,61	41,91	distal	conventional	optical
4648	90,92	86,98	mesial	conventional	optical
4648	188,22	80,77	distal	conventional	optical
4649	365,37	258,55	mesial	conventional	optical
4649	210,2	90,68	distal	conventional	optical
4712	258	49,9	mesial	digital	SEM
4712	446	172	distal	digital	SEM
4713	139	76,9	mesial	digital	SEM
4713	123	58,2	distal	digital	SEM
4714	93,4	56,8	mesial	digital	SEM
4714	293	128	distal	digital	SEM
4715	167	51,4	mesial	digital	SEM
4715	196	111	distal	digital	SEM
4716	158	35,4	mesial	digital	SEM
4716	368	209	distal	digital	SEM
4717	94	39,4	mesial	digital	SEM
4717	83,1	11,8	distal	digital	SEM
4718	344	181	mesial	digital	SEM
4718	298	174	distal	digital	SEM
4719	211	93,8	mesial	digital	SEM
4719	241	116	distal	digital	SEM
4720	120	46	mesial	digital	SEM
4720	174	106	distal	digital	SEM
4721	207	66	mesial	digital	SEM
4721	388	278	distal	digital	SEM

4722	63,3	58,1	mesial	digital	SEM
4722	146	56,8	distal	digital	SEM
4723	88,9	0	mesial	digital	SEM
4723	244	79,2	distal	digital	SEM
4724	339	88,3	mesial	digital	SEM
4724	494	278	distal	digital	SEM
4725	74,9	47,4	mesial	digital	SEM
4725	169	74,5	distal	digital	SEM
4726	99	48,3	mesial	digital	SEM
4726	195	119	distal	digital	SEM
4727	75,4	69,3	mesial	digital	SEM
4727	173	122	distal	digital	SEM
4728	270	194	mesial	digital	SEM
4728	297	155	distal	digital	SEM
4729	388	184	mesial	digital	SEM
4729	318	109	distal	digital	SEM
4730	108	22,8	mesial	digital	SEM
4730	147	76,2	distal	digital	SEM
4731	148	20,1	mesial	digital	SEM
4731	225	108	distal	digital	SEM
4712	232,94	57,42	mesial	digital	optical
4712	367,37	150,55	distal	digital	optical
4713	60,12	50,3	mesial	digital	optical
4713	85,22	49,47	distal	digital	optical
4714	82,36	46,3	mesial	digital	optical
4714	241,75	114,47	distal	digital	optical
4715	not available	not available	mesial	digital	optical
4715	not available	not available	distal	digital	optical
4716	118,04	50,73	mesial	digital	optical
4716	298,81	181,93	distal	digital	optical
4717	50,07	44,38	mesial	digital	optical
4717	117,94	53,85	distal	digital	optical
4718	352,46	183,62	mesial	digital	optical
4718	293,94	146,86	distal	digital	optical
4719	192,11	25,23	mesial	digital	optical
4719	240,33	131,13	distal	digital	optical
4720	115,36	40,72	mesial	digital	optical
4720	187,76	121,19	distal	digital	optical
4721	135,8	75,1	mesial	digital	optical
4721	347,79	230,02	distal	digital	optical
4722	72,4	29,09	mesial	digital	optical
4722	138,69	87,62	distal	digital	optical
4723	152,4	39,45	mesial	digital	optical
4723	195,84	103,12	distal	digital	optical
4724	128,89	61,41	mesial	digital	optical
4724	380,61	236,76	distal	digital	optical
4725	62,24	28,34	mesial	digital	optical
4725	173,08	83,63	distal	digital	optical
4726	79,28	46,92	mesial	digital	optical
4726	152,1	110,61	distal	digital	optical
4727	not available	not available	mesial	digital	optical
4727	not available	not available	distal	digital	optical

4728	256,08	175	mesial	digital	optical
4728	264,58	175,57	distal	digital	optical
4729	458,39	196	mesial	digital	optical
4729	275,52	96,41	distal	digital	optical
4730	124,4	32,61	mesial	digital	optical
4730	127,31	72,91	distal	digital	optical
4731	73,06	29,76	mesial	digital	optical
4731	260,5	107,72	distal	digital	optical

Additional experiments

specimen nr.	MG		AMD	
	mesial	distal	mesial	distal
5990	78,91	61,7	130,6	280,11
5991	not available	186,5	not available	207,5
5992	92,4	90	not available	163,1
5993	257,5	91,2	375,3	132,4
5994	364,3	50	735,4	148,2
5995	30,64	71,2	82,8	124,8
5996	87,5	164,4	127,8	180,6
5997	102,8	64	136,7	73,7
5998	117,5	69,1	120,7	72,5
5999	42,69	51	59,6	256,8
